# Presentation of the Subject of State Control of Tuberculosis (Concluded)

**Published:** 1897-11

**Authors:** John M. Parker

**Affiliations:** Member of the Massachusetts Cattle Commission, Haverhill, Mass.


### PRESENTATION OF THE SUBJECT OF STATE CONTROL OF TUBERCULOSIS.[1]

By John M. Parker, D.V.S.,
MEMBER OF THE MASSACHUSETTS CATTLE COMMISSION, HAVERHILL, MASS.

(Concluded from page 615.)

But the work of the Board does not end here. The law says (Section 45 of Chapter 491, of 1894, as amended by Section 10

[1] Read before the Thirty-fourth Annual Meeting of the United States Veterinary Medical Association, Nashville, Tenn., September, 1897.

of Chapter 496 of 1895): "When the Board of Cattle Commissioners or any of its members, by an examination of a case of contagious disease among domestic animals, become satisfied that the public good requires it, such Board of Commissioners shall cause such animal or animals affected therewith to be securely isolated, or shall cause it or them to be killed without appraisal or payment, provided, however, that whenever any cattle condemned as afflicted with the disease of tuberculosis are killed under the provisions of this section, the full value thereof at the time of condemnation, not exceeding the sum of sixty dollars for any one animal, shall be paid to the owner thereof out of the treasury of the Commonwealth, if such animal has been owned within the State six months continuously prior to its being killed."

There was nothing in this law to prevent an owner having his herd tested by private test, if he so desired. If the test was made by a regular veterinarian, the State could only accept it, appraise, and destroy the cattle and grant the owner compensation. This was an ideal method for both the unscrupulous owner and veterinarian. By this method there were no restrictions placed on the owner—he could test as many or as few animals as he desired. Many times owners would have their herds tested without any idea of attempting to keep them free from disease. When the reacting animals were destroyed others were bought without test and often placed in the same barn without any attempt at cleansing or disinfection. Such a method of doing business was neither common sense nor business-like. There was little use in the State paying for cattle that reacted to a private test unless the owner really desired to clean up his herd, and meant to stick to it once he began it. This is a point of a good deal of practical importance. The business man and the capitalist; the men who make a business gain out of the sale of milk from tuberculin-tested cows; the men who run their own milk-route, who have their own customers, and who get from two to three cents more per quart because of the test; such men naturally wish their cows tested, and would naturally keep it up as a business investment. Then, again, the men who raise their own stock—and who wish to get rid of disease for the sake of their own herds, in which they are interested—would also be likely to keep up the work in one way or another, for they think that if they are once rid of the disease they stand a fair chance of keeping free from it. They have got an object in view; they aim at something definite.

On the other hand, the dairyman who ships his milk to the contractor, who gets the contractor's price, who has the surplus question to deal with, who sells his cow when she runs dry and buys fresh cows to replace the old, who has to buy frequently and test every cow he buys, and run chances at that—what encouragement has he to keep up the work? There is no profit in it for him; he has no increased price for his milk; there is simply added expense without increased gain. Is it any wonder, then, although at first he may be enthusiastic, yet, after a time, he loses interest, and, finally, through want of enthusiasm and want of encouragement, he is apt to become negligent and introduce cows into his herd that have not been tested? And this carelessness and indifference, of course, is liable at any time to undo any good that had already been done.

Realizing something of this, and feeling that many farmers having their herds tested by private test did not recognize their responsibility in the matter, a letter was sent to each individual, calling his attention to the law allowing him compensation "not exceeding the sum of $60" for animals killed as tuberculous, provided such person shall not have, prior thereto, in the judgment of the Cattle Commissioners, by wilful act or neglect contributed to the spread of tuberculosis; but such decision on the part of the commissioners shall not deprive the owner of the right of arbitration as hereinafter provided." The letter then continued: "If an owner does not thoroughly disinfect and cleanse his barn, or if, after having had cattle taken and paid for by the State, he introduces untested animals into his herd, he has through his neglect contributed to the spread of tuberculosis, and, therefore, under this section, forfeits his right to compensation for such tuberculous animals as may hereafter be found in his herd."

After having had their attention called to the matter, one would naturally suppose that farmers who had gone to the expense of having a private test made, costing them anywhere from $10 to $100 and over, would take interest enough in the matter to cleanse their barns, especially after being warned of the penalty of neglect; but on investigation it was found to be the exception rather than the rule when any cleansing, even of the simplest nature, was done.

The Board then decided to send around a special agent, whose duty it would be to call on each individual who had had his herd tested, see whether the barn had been attended to, and report to the Board. This was done, and this work has been specially productive of much good. Up to the end of August 530 barns, where private tests have been made, had been visited; of these 530 barns it was found that only 42 had been cleansed and disinfected, and in 488 absolutely nothing had been done in the way of cleansing. Out of the 530 only 95 were respectably clean, and 435 were filthy. The usefulness of this work is well known by the results following these visits. By the end of August a second visit had been made of 158 barns where the owner had previously been found negligent. Of these 158 barns visited the second time 134 were found cleansed and disinfected, and in only 22 had nothing been done. These 22 were in a dirty condition, several of them being absolutely filthy. Such work as this is exceedingly important, not only because of the practical amount of good it does in removing infectious material from the barns, but in an educational way as well. This, I believe, is the key to the whole question, and it is surely something that no intelligent man can reasonably object to.

During the early months of the year the testing of herds by private test, where the owner seemed to care for little except how to get a good appraisal for his cattle, became so frequent that in June the Legislature passed a law to the effect that "no person having animals tested with tuberculin shall be entitled to compensation from the treasury of the Commonwealth for any animals that react to the tuberculin-test, unless such testing be done by the State Board of Cattle Commissioners, or their authorized agents acting as such at the time of the test, and such testing shall be subject to the supervision and control of the State Board of Cattle Commissioners. This act shall take effect upon its passage. (Approved June 10, 1897)."

The immediate effect of this law was to shut off indiscriminate, irresponsible testing. The State is willing to assist an owner to purge his herd from disease if the owner is in earnest, but before it will undertake to assist it requires him to sign a written agreement that he will observe the sanitary regulations prescribed by the Board, and that he will not introduce any animals into his herd without first subjecting them to the tuberculin-test. In this way herds are only tested where the owner is not only willing but anxious to keep up the work, and, of course, under these circumstances there is a much better prospect of the work resulting in permanent improvement.

But many times where there is not only enthusiasm but unlimited capital behind the endeavor to rid a herd of disease, even then it is not always an easy matter. About eighteen months or two years ago a number of herds in Massachusetts were tested with tuberculin, under promise from the owners to observe certain conditions. Among those tested was one belonging to Mr. H., consisting of 81 animals. This herd was carefully tested and the figures submitted to the Board, and 28 animals considered certainly diseased by the Board were condemned and destroyed, and a number were held for retest. The animals held for retest were kept in a separate pasture and were not allowed to mingle with the rest of the herd. Of these, 13 animals were retested, condemned by the Board, and killed; the others passed as sound. Before these cattle were allowed to join the herd an attempt was made to disinfect the barn as thoroughly as possible.

In October, 1896, sixteen months after the first test, the farm was again visited, and one lot of 50 head kept in the new barn were tested; of this number 29 were of the original lot, and 18 were Vermont cattle that had been tested carefully before being shipped to the farm, especial pains being taken to secure only healthy animals; 3 others, not tested, were introduced into the herd on or about September 30, and at that time Mr. H. notified the Board, and it was expected that the entire herd would be retested immediately. However, because of the difficulty in securing sufficient tuberculin, a delay of a few weeks occurred, and it was not until October that two members of the Board visited the herd and tested the lot. Of the 50 animals tested, 27 reacted, were condemned and killed, 25 of which proved to be tuberculous upon post-mortem examination.

About two months later the remaining 28 animals in the old barn, which had been kept entirely separate, were tested and 16 gave well-marked reactions. The barns were disinfected and the 16 cows, being mostly dry cows and springers, were isolated on one side of the barn, two partitions being placed between them and the other cattle. They stayed here until March 5th, when they were again tested, and at this time they gave 14 well-marked reactions. The temperature of the other two cows at the second test read as follows:

No. 129. 1st test, Jan. 8, 101°, Jan. 9, 101°, 104.4°, 103.4°, 103.2°.
2d test, Mar. 5, 101°, Mar. 6, 101.3°, 101.3°, 101.3°, 103°, 99.4°.
No. 5468. 1st test, Jan. 8, 101°, Jan. 9, 104°, 105°, 105.1°, 104.4°.
2d test, Mar. 5, 101°, Mar. 6, 101.4°, 101.4°, 102.2°, 102.2°, 102.2°.

Both were killed, and on post-mortem examination proved to be diseased. The barns were again disinfected with bichloride of mercury, 1 : 800, and whitewashed later.

On May 1, 1897, the entire herd of 84 head were again tested, and this time 15 animals, or 17 per cent., gave reactions, and were killed. The post-mortem examinations showed these animals to have been only very slightly diseased. The barns have been twice disinfected during the summer, and another test will probably be made in October. After every test, when cattle have been condemned, the herd has invariably been replenished with tested cows from Vermont.

A second herd belonging to Mr. E. is another good illustration of the difficulty in thoroughly disinfecting barns. After being first tested and cleaned up in October, 1894, this herd was retested on March 17 and 18, 1896. There were 80 animals in the herd, 78 of which were tested, 2 animals having a temperature too high at the time to be injected. 14 animals reacted, or showed a suspicious rise in temperature; they were immediately separated from the remainder of the herd, were again retested on June 19th and 20th, at which time 2 were released, 3 were condemned, and 9 were continued in quarantine. On October 7th, 8th, and 9th these 9 quarantined animals were tested and condemned.

On August 16 and 17, 1896, 64 of the original herd and 1 animal which had been introduced recently were tested. None of the original animals reacted to the test, while the animal which had been introduced from the outside reacted, was condemned, and, upon post-mortem, was found to be tuberculous.

In the meantime a new barn had been built and was used as the home barn. The old barns were disinfected as thoroughly as possible and the young stock and dry cows were housed in them, the fresh cows being brought to the new barn as they came in.

In the month of August, 1897, exactly one year after the previous test, the herd was again tested. At this time there were 154 animals at the home farm. These were all tested, 22 were condemned and killed as tuberculous, and 32 were released. 5 out of 54 at the second farm were condemned, and 1 out of 58 at the third farm was also condemned. All these cows were found to be diseased, the disease being very slight and confined entirely to the bronchial and mediastinal glands.

It is but fair to state that another of the reacting animals was a registered bull, "Sir Michael." He had been kept isolated from the rest of the herd, except when being used for service, since March, 1896, when he first reacted. At that time the temperatures were: 8 P.M., 101°; 6 A.M., 101°, 101.2°, 103.4°, 104.4°, 104.3°, 104.2°, 104.3°, 104.2°, 103°. On June 13, 1896: 9.30

P.M., 101.1°; 7.30 A.M., 102.3°, 103.1°, 103.1°, 103°, 103°, 101.3°. On August 18, 1896: 7 P.M., 101.2°; 5 A.M., 101.1°, 101°, 102.2°, 103.4°, 104°, 103.4°, 103.4°. On August 10, 1897: 8 P.M., 101°, 101.1°; 7 A.M., 101.2°, 103.1°, 104.3°, 105°. This bull has been used for service on nine cows out of the twenty-two that have since reacted and were killed, one of the cows had been served as recently as July 21, 1897, or just about four weeks before the last test was made. The fact that none of the cows showed any traces of tuberculosis except in the bronchial and mediastinal glands would seem to exclude the bull as a possible source of infection so far as sexual intercourse was concerned in any of these cases.

In studying the problem of State control I should like to call your attention to the principles of sanitary science as applied in the United States and European countries to the suppression and prevention of contagious disease. A few years ago, at the time of the latest cholera-scare, the seaports of this country were all guarded and a strict quarantine of all vessels coming to this country from infected ports was enforced. Most European countries took the same precaution. Great Britain, however, proved an exception to the rule. Her ports were open to the world, vessels of all nationalities went in and out, and tourists and travellers from all countries had the right to go and come as they pleased. Great Britain relied on the excellence of her internal sanitary arrangements to combat the disease. If she had placed all vessels from infected ports in quarantine the loss of the country would have been tremendous. The traffic between Great Britain and other countries was too great, and the number of vessels entering her ports from infected countries too numerous to permit of it. The disturbance to traffic would have been enormous, and, in the opinion of the authorities, the gain would not have been commensurate with the probable loss. Other means, therefore, had to be relied on to control and check the spread of the disease. In the same way the difficulties in the way of the total and permanent exclusion of the tubercle bacillus are too great to permit of our relying on these means alone. We must not only attempt to control the breeding-places of the bacillus, and lessen the number that are mixed with the dust in infected premises, but we must look to the perfecting of internal sanitary arrangements, so that individuals may not become such easy victims of the disease, and, by using all the means in our power, and by constant and continual effort, the existing conditions may be bettered, so that in time we may reach some measure of success without the great expense involved in the total destruction of all animals that react to the test.

In Massachusetts during the year 1895 there were 1549 cattle tested by private request, representing one hundred and nine entire herds. Of this number 433, or 27.95 per cent., were condemned. In 1896 there were 2021 cows tested by private request, representing two hundred and eighty-one entire herds. Of this number 1098, or 54.32 per cent., were condemned. This year, up to July, full returns had been received from 1810 cows, representing one hundred and fifty-three entire herds. Of this number 911, or 52.54 per cent., were condemned. This percentage includes four herds recently cleaned up in the western part of the State. These four herds include 96 animals, only 3 reacting, making a percentage of 3.1. It will be interesting to see how these tests stand a year from now.

This work is different from the work of the local inspection in that the inspectors only pick out such animals as show some physical evidence of disease, while this work represents the testing of entire herds scattered at haphazard over the Commonwealth. If this is taken, then, as any sort of guide to the number of diseased cows in the State, there are, roughly, over half of the milk-cows in the State that would react to tuberculin.

During the present year the average price actually paid for cattle condemned for tuberculosis has been $35.80 per animal. The number of neat cattle over one year old assessed in the Commonwealth in 1896 was 212,601, and probably about seventeen-twentieths of these were milk-cows. It does not require much calculation, then, to find out that if all cows were tested, and all those reacting were killed off, it would cost the State about $3,500,000 to pay for the cows killed, not to mention the cost of the executive portion of the work.

The authorities in Pennsylvania and elsewhere have placed themselves on record as being of the opinion that, from the enormous cost of the work, the testing of every cow in their various districts is impractical. Massachusetts is of the same opinion. She believes that the danger to man from bovine tuberculosis can be lessened by the adoption of broader methods in control work. For example, I believe that the entire work would be benefited, both as to expedition and thoroughness, if the State was divided up into veterinary districts, each district in charge of an experienced, qualified veterinarian, who would be responsible to the commission for the supervision of the herds and the slaughter-houses established by the

State or city in his district. It is time, I believe, that the country slaughter-house was done away with. It is time the State took the matter up, and I believe that an opportune moment for the change has come. Greater economy must be used in the disposal of carcasses of animals that react to the test, especially as over 90 per cent. of the animals condemned seem to have only localized tuberculosis. I believe that State slaughter-houses could be established; that stalls in these slaughter-houses could be rented to local parties. This would greatly simplify the inspection service, and the dirty, filthy country slaughter-house would have to go; and there would be this further advantage that in State or municipal slaughter-houses, under a proper system of inspection, much of the meat at present condemned to the rendering-tank could safely be used for food. This is a matter that I hope to see taken up and acted on at an early date.

---

Madison, Wis., receives milk from a herd of thirty Jersey grades. As soon as drawn the milk is carried into a screen-protected power-house, and run through a separator, not to collect the cream, but for cleansing, and the cream and milk come together again in a large can, where they are thorougly stirred and then moved across the room to a surface-cooler, a metallic surface of nine feet square, over the back of which runs a stream of ice-water, while the milk runs down the face of it into cans. This reduces the temperature from 85° to 45°. From these cans the cool milk is elevated and poured into a moving vat with spring-faucets. The vat travels on a narrow railway over a box in which are placed the delivery cans of glass, quarts and pints, the stoppers being coated with hot paraffin. When filled these are placed in cases thirty inches long and ten inches high, and covered with finely chopped ice. This milk sells in Madison for five cents a quart, and will keep from twenty-four to forty-eight hours longer than when treated in the ordinary way.

A recent test of a six-year-old bay gelding, sired by a thoroughbred horse, the dam by Onward, travelled a distance of thirty miles on the Lancaster pike, leading from Philadelphia, without being touched with a whip or urged with a line, in two hours and thirty-nine minutes. The first ten miles in fifty-six minutes, the second in forty-four minutes, and the third in fifty-nine minutes. This was at a trotting gait. This horse recently ran a half mile in forty-eight and one-quarter seconds.

